# A Note on Modelling Bidirectional Feedback Loops in Mendelian Randomization Studies

**DOI:** 10.1007/s10519-024-10183-0

**Published:** 2024-05-31

**Authors:** Liang-Dar Hwang, David M. Evans

**Affiliations:** 1 Institute for Molecular Bioscience, The University of Queensland, Brisbane, QLD Australia; 2 The Frazer Institute, The University of Queensland, Woolloongabba, QLD 4102 Australia; 3 MRC Integrative Epidemiology Unit, University of Bristol, Bristol, UK

**Keywords:** Mendelian randomization, Bidirectional Mendelian randomization, Structural equation modelling, Causation, Feedback loops, Reciprocal causation

## Abstract

Structural equation models (SEMs) involving feedback loops may offer advantages over standard instrumental variables estimators in terms of modelling causal effects in the presence of bidirectional relationships. In the following note, we show that in the case of a single “exposure” and “outcome” variable, modelling relationships using a SEM with a simple bidirectional linear feedback loop offers no advantage over traditional instrumental variables estimators in terms of consistency (i.e. both approaches yield consistent estimates of the causal effect, provided that causal estimates are obtained in both directions). In the case of finite samples, traditional IV estimators and SEM exhibited similar power across many of the conditions we examined, although which method performed best depended on the residual correlation between variables and the strength of the instruments. In particular, the power of SEM was insensitive to the residual correlation between variables, whereas the power of the Wald estimator/2SLS improved (deteriorated) relative to SEM as the magnitude of the residual correlation increased (decreased) assuming a positive causal effect of the exposure on the outcome. The power of SEM improved relative to the Wald estimator/2SLS as the instruments explained more residual variance in the “outcome” variable.

**Supplementary Information:**

The online version contains supplementary material available at 10.1007/s10519-024-10183-0.

Mendelian randomization (MR) is an epidemiological method that uses genetic variants as instrumental variables to inform on potential causal relationships in observational data (Smith and Ebrahim, 2003). MR studies often employ two-stage least squares in the case of one sample MR, where the exposure and outcome are measured in the same group of individuals (Anderson and Rubin 1950; Basmann 1957; Sargan 1958), or the Wald ratio, in the case of two sample MR, where the exposure and outcome are measured in different samples (Wald 1940), to estimate causal effects. MR can also be performed using maximum likelihood structural equation modelling (Maydeu-Olivares et al. 2019) although it has not been common to do so in the literature to date (Castro-de-Araujo et al. 2023; Minica et al. 2018). Nevertheless, it is possible that structural equation modelling may offer some advantages over traditional instrumental variables estimators in the presence of bidirectional feedback loops between variables (i.e. when the “exposure” and “outcome” variables reciprocally affect each other) since these structures can be easily accommodated within a structural equation modelling framework, whereas traditional estimators do not model these relationships explicitly.

In the following note, we show analytically via asymptotic theory that in the case of a single “exposure” and “outcome” variable, modelling bidirectional relationships using a SEM with a simple linear feedback loop surprisingly offers no advantage over traditional instrumental variables estimators in terms of consistency (i.e. both approaches yield consistent estimates of the causal effect, provided that causal estimates are obtained in both directions). However, we then show via simulation in large, but finite samples, that the relative power of the approaches depends on instrument strength (in particular the amount of residual variance explained in the “outcome”), and the strength of the residual covariance between the variables.

## The Population Model

**Fig. 1:**
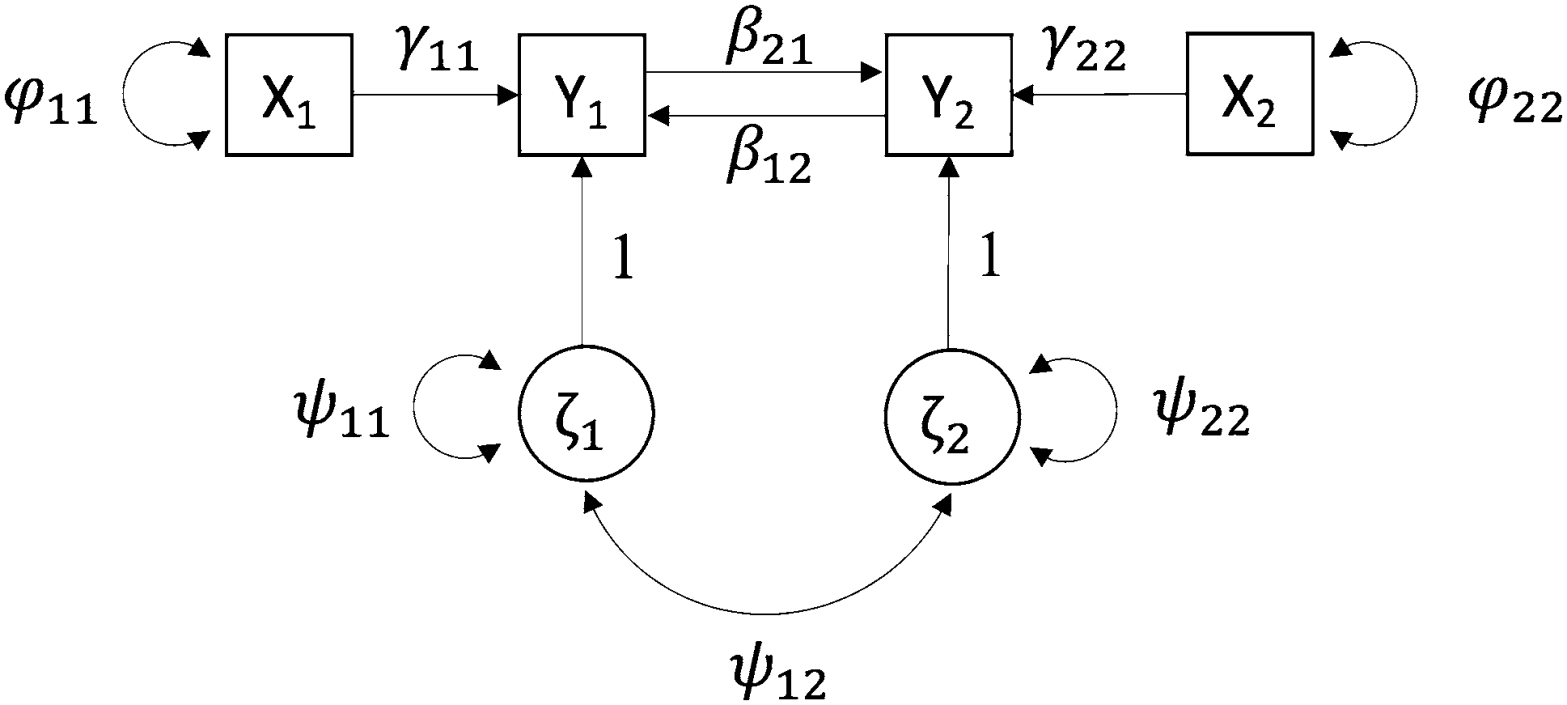
A path diagram illustrating a SEM involving feedback loops parameterized using the LISREL model. The exogenous variables x_1_ and x_2_ serve as genetic instruments for the endogenous y_1_ and y_2_ variables respectively. Residual variation affecting y_1_ and y_2_, which may include genetic and environmental sources, are modelled as correlated disturbance terms (ζ_1_, ζ_2_)

We assume that the relationship between two endogenous variables y_1_ and y_2_ is given by the SEM in Fig. 1 where we have used the LISREL notation to describe the underlying population model (Bollen 1989) (NB. The following results can also be obtained using other frameworks including McArdle and McDonald’s RAM approach (McArdle and McDonald 1984)). The y_1_ and y_2_ variables reciprocally influence each other (as indexed by the paths β_21_ and β_12_) and are instrumented by exogenous variables x_1_ and x_2_ respectively. We note that in order for the model to be identified and the reciprocal causal paths uniquely estimated, it is necessary to instrument both y_1_ and y_2_ variables. The strength of each instrument is indexed by the path coefficients γ_11_ and γ_22_. The endogenous y_1_ and y_2_ variables are also a function of disturbance terms (ζ_1_, ζ_2_) which comprise the sum total latent residual genetic and environmental factors that affect y_1_ and y_2_ respectively. These disturbances may or may not be correlated and the degree of sharing is quantified by the covariance parameter (ψ_12_) that represents the amount of latent confounding between y_1_ and y_2_. We assume linearity in the relationships between exogenous and endogenous variables, no effect modification, and that the disturbance terms of the endogenous variables are distributed multivariate normal in the population.

## Model Specification in LISREL Matrix Notation

The model in Fig. 1 can also be written in matrix notation using the LISREL model:

$$\text{y}=\text{B}\text{y}+{\Gamma x}+\upzeta$$

where.

$$\text{y}=\left[\begin{array}{c}{y}_{1}\\ {y}_{2}\end{array}\right]$$;﻿ 2 × 1 vector of observed dependent variables.

$$\text{x}=\left[\begin{array}{c}{x}_{1}\\ {x}_{2}\end{array}\right]$$; 2 × 1 vector of (genetic) instruments.

$$\text{B}=\left[\begin{array}{cc}0& {\beta }_{12}\\ {\beta }_{21}& 0\end{array}\right]$$; 2 × 2 coefficient matrix for the reciprocal causal effects of the y variables.

$$\Gamma =\left[\begin{array}{cc}{\gamma }_{11}& 0\\ 0& {\gamma }_{22}\end{array}\right]$$; 2 × 2 coefficient matrix for the SNP effects of the x on y variables.

$$\upzeta =\left[\begin{array}{c}{\upzeta }_{1}\\ {\upzeta }_{2}\end{array}\right]$$; 2 × 1 vector of residual disturbance terms.

Without loss of generality, we assume that x and y are measured in deviation form. Noting that:

$$\text{y}=\text{B}\text{y}+{\Gamma x}+\upzeta$$

and so

$$\text{y}={\left(\text{I}-\text{B}\right)}^{-1}\left({\Gamma x}+\upzeta \right)$$

the covariance matrix of y is given by (Bollen 1989):

$${\sum}_{xx} \; = \;E\left( {{\text{yy}}^{\prime} } \right)\, = \,E[\left( {{\text{I}} - {\text{B}}} \right)^{ - 1} \left( {\Gamma {\text{x}} + {\upzeta }} \right) \left( {\left( {{\text{I}} - {\text{B}}} \right)^{ - 1} \left( {\Gamma {\text{x}} + {\upzeta }} \right)} \right)^{\prime} ]$$

$$= E\left[ {\left( {{\text{I}} - {\text{B}}} \right)^{ - 1} \left( {\Gamma {\text{x}} + {\upzeta }} \right) \left( {{\text{x}}^{\prime} {\Gamma }^{\prime} + {\upzeta }^{\prime} } \right)\left( {{\text{I}} - {\text{B}}} \right)^{ - 1^{^{\prime} } } } \right]$$

$$= \left( {{\text{I}} - {\text{B}}} \right)^{ - 1} (E\left( {\Gamma {\text{xx}}^{\prime} \Gamma^{\prime} } \right) + E\left( {\Gamma {\text{x}\zeta }^{\prime} } \right) + E\left( {{\zeta x}^{\prime} \Gamma^{\prime} } \right) + E({{\zeta \zeta }}^{\prime} ))\left( {{\text{I}} - {\text{B}}} \right)^{ - 1^{^{\prime} } }$$

$$= \left( {{\text{I}} - {\text{B}}} \right)^{ - 1} (\Gamma {\Phi }\Gamma^{\prime} + {\Psi })\left( {{\text{I}} - {\text{B}}} \right)^{ - 1^{^{\prime} } }$$

where

$$\Phi =\left[\begin{array}{cc}{\phi }_{11}& 0\\ 0& {\phi }_{22}\end{array}\right]$$ is the covariance matrix of x.

$$\Psi =\left[\begin{array}{cc}{\psi }_{11}& \\ {\psi }_{21}& {\psi }_{22}\end{array}\right]$$ is the covariance matrix of residual errors ζ (lower elements only shown).

Similarly, the covariance matrix of x and y is given by:

$${\sum}_{xy} = E\left( {{\text{xy}}^{\prime} } \right) = E[{\text{x}}\left( {\left( {{\text{I}} - {\text{B}}} \right)^{ - 1} \left( {\Gamma {\text{x}} + {\upzeta }} \right)} \right)^{\prime} ] = {\Phi }\Gamma^{\prime} ({\text{I}} - {\text{B}})^{ - 1^{^{\prime} } }$$

and as noted above:

$${\Sigma }_{xx} = E\left( {{\text{xx}}^{\prime} } \right) = {\Phi }$$

## Consistency of the Wald Estimator for Two Dependent Variables

It is possible to derive estimates of the causal parameters ${\beta }_{12}$ and ${\beta }_{21}$ using the Wald ratio (or alternatively two-stage least squares). This means that the MR analysis needs to be run “both ways”. In other words, first x_1_ is used to instrument the causal effect of y_1_ on y_2_ and the parameter ${\beta }_{21}$ is estimated. Then in a separate analysis, x_2_ is used to instrument the causal effect of y_2_ on y_1_ and the parameter ${\beta }_{12}$ is estimated. The Wald estimator of the causal effect of y_1_ on y_2_ is given by:

$${\widehat{\beta }}_{21}^{*}=\frac{\text{cov}({x}_{1}, {y}_{2})/\text{var}({x}_{1})}{\text{cov}({x}_{1}, {y}_{1})/\text{var}({x}_{1})}=\frac{\text{cov}({x}_{1}, {y}_{2})}{\text{cov}({x}_{1}, {y}_{1})}$$

where cov and var refer to the sample covariances and variances of the observed **x** and **y** variables. Taking the probability limit of this estimator:

$$\text{plim}\left({\widehat{\beta }}_{21}^{*}\right)=\text{plim}\left[\frac{\text{cov}({x}_{1}, {y}_{2})}{\text{cov}({x}_{1}, {y}_{1})}\right]=\frac{\text{COV}({x}_{1}, {y}_{2})}{\text{COV}({x}_{1}, {y}_{1})}=\frac{{\phi }_{11}{\gamma }_{11}{\beta }_{21}(1-{\beta }_{21}{\beta }_{12})}{{\phi }_{11}{\gamma }_{11}(1-{\beta }_{21}{\beta }_{12})}={\beta }_{21}$$

shows that the Wald estimator ${\widehat{\beta }}_{21}^{*}$ is a consistent estimator of the population causal parameter ${\beta }_{21}$, where COV refers to the population covariance. A similar derivation shows that ${\widehat{\beta }}_{12}^{*}$ is a consistent estimator of the population causal parameter ${\beta }_{12}$. Thus, SEMs such as the one depicted in Fig. 1 that model the bidirectional relationships between two observed variables in the form of a simple linear feedback loop (and therefore themselves produce consistent estimates of ${\beta }_{12}$ and ${\beta }_{21}$), do not have an advantage over the standard Wald Estimator in terms of consistency (interestingly we show in the Supplementary Note that in the case of correlated instruments (i.e. ${\phi }_{12}$ = ${\phi }_{21}$  ≠ 0), the Wald estimator is no longer a consistent estimator of the causal effect parameters).

## Finite Sample Properties and Power Comparison

To investigate the finite sample properties of both estimators, we simulated data assuming the model displayed in Fig. 1. Both dependent variables y_1_ and y_2_ and the dosages at the two “additive” SNPs (x_1_ and x_2_) were simulated to have a variance of one. We varied the strength of the causal effect size (${\beta }_{21}$ = 0, 0.1, 0.5; ${\beta }_{12}$ = 0.1, − 0.1, 0.5), the instrument strength (${\gamma }_{11}$ = 0.1, 0.3; ${\gamma }_{22}$ = 0.1, 0.3, 0.5), the overall correlation between y_1_ and y_2_ (r(y_1_, y_2_) = 0.1, 0.3, 0.5), and sample size (N = 1000 to 10,000, in increments of 1000). Each condition was simulated 1000 times and power/type I error rate was calculated as the number of simulations in which the effect of interest was detected (α < 0.05) divided by 1000. We calculated the Monte Carlo standard errors and 95% confidence intervals for causal effect estimates, type I error rate, coverage and power. We fit the Wald estimator to each replicate estimating the causal effect ${\beta }_{21}$ by the ratio of the linear regression coefficient of y_2_ on x_1_, and y_1_ on x_1_, as well as its standard error including second order terms (Thomas et al. 2007). We evaluated significance using a standard normal distribution. Similar patterns of results were obtained when using two-stage least squares instead of the Wald ratio. We also fit a SEM to the data that had the same form as in Fig. 1 (i.e. the SEM is the same as the population generating model for the data), and computed the maximum likelihood estimate for ${\beta }_{21}$ and ${\beta }_{12}$, their standard error, and significance via log-likelihood ratio test.

## Results and Discussion

**Fig. 2:**
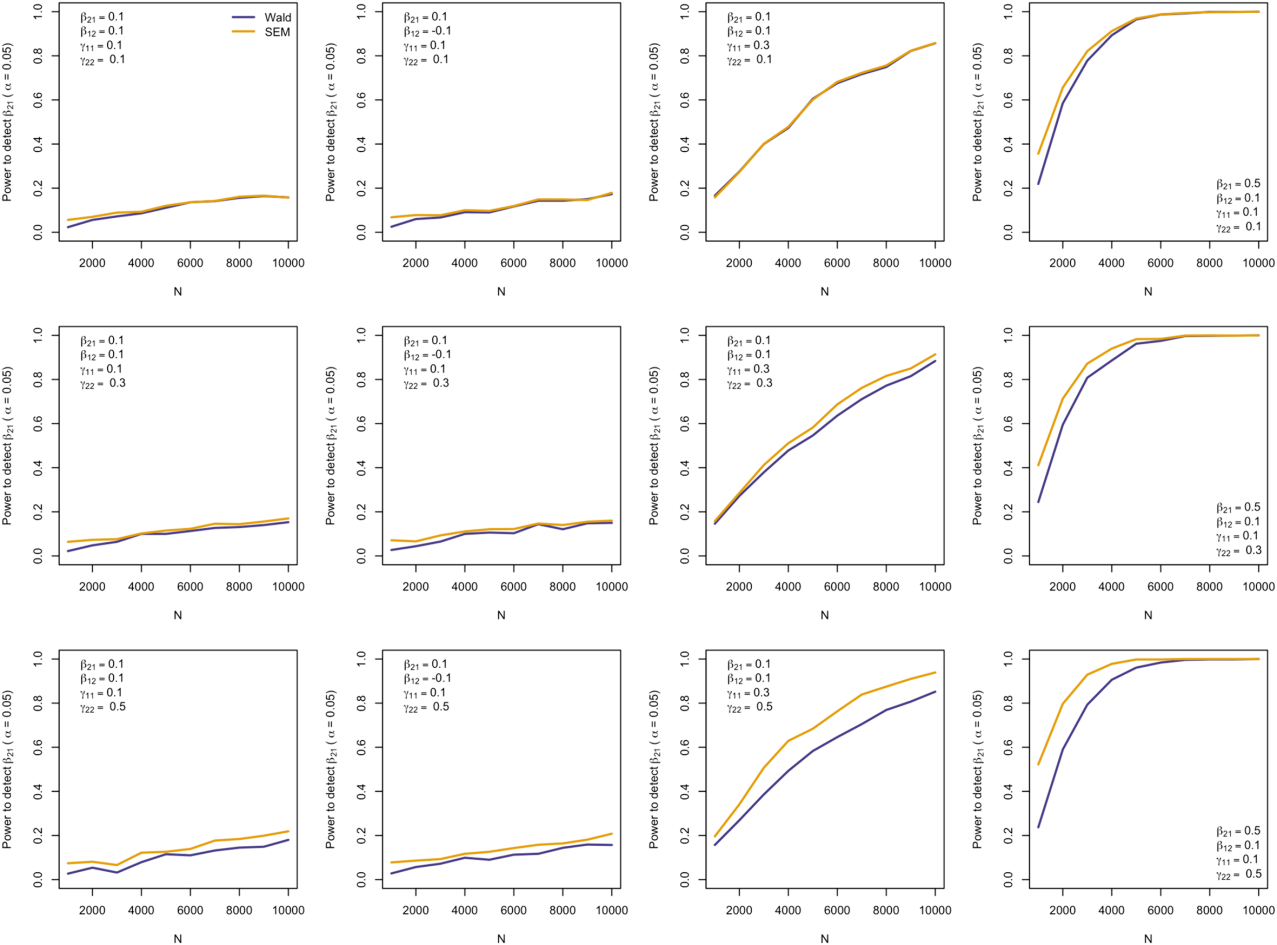
Power to detect the causal parameter ${\beta }_{21}$ as a function of ${\gamma }_{22}$ (i.e. the variance in y_2_ explained by x_2_). The parameter ${\gamma }_{22}$ is set to ${\gamma }_{22}$ = 0.1 in the top row, ${\gamma }_{22}$ = 0.3 in the middle row, and ${\gamma }_{22}$ = 0.5 in the bottom row. The parameters used in each simulation are displayed in the legend in the top left (or bottom right) corner of each graph. Power does not vary as ${\gamma }_{22}$ increases when using the  Wald method, but increases when using maximum likelihood structural equation modelling

**Fig. 3:**
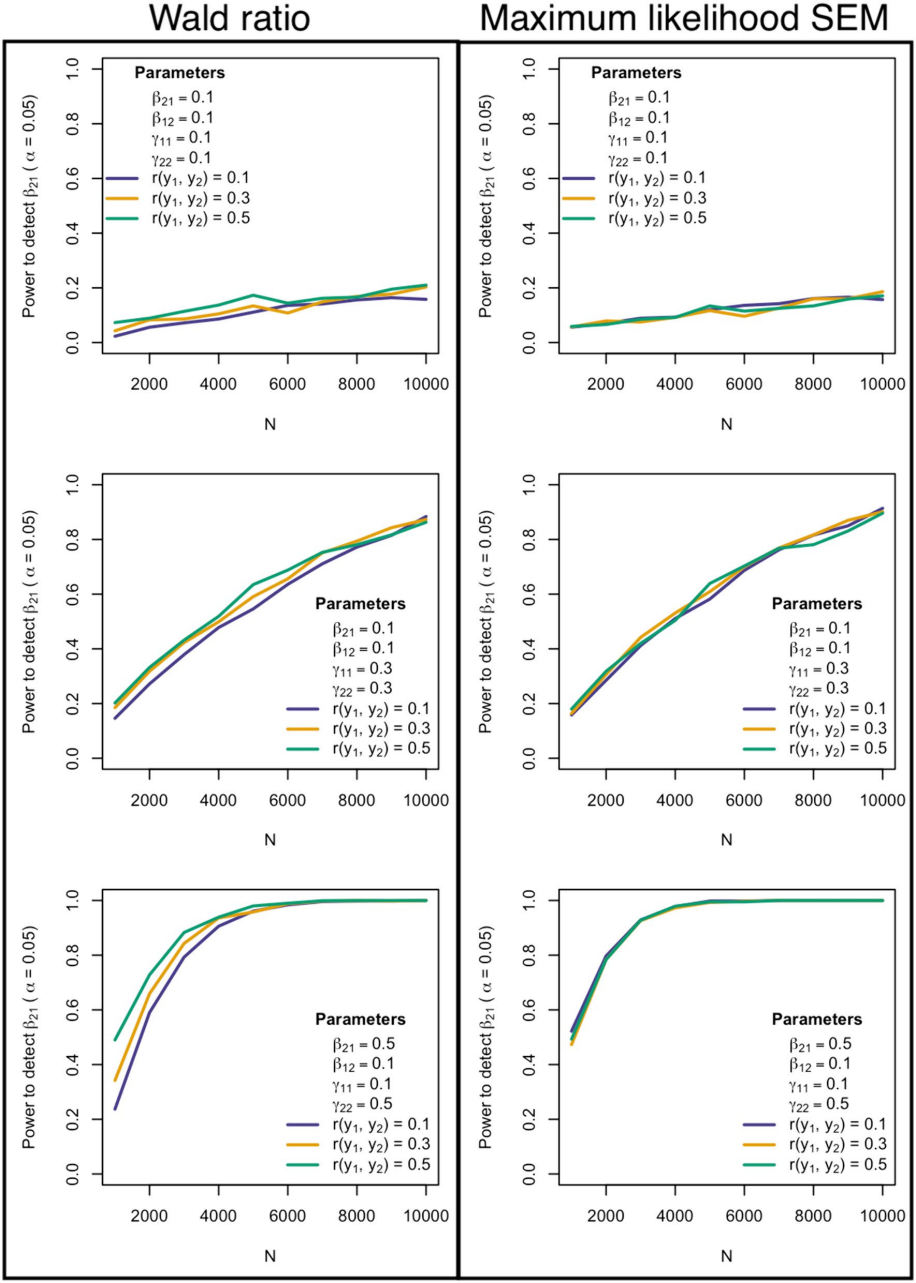
Power to detect the causal parameter ${\beta }_{21}$ as a function of the correlation between variables (i.e. r(y_1_, y_2_)) for the Wald ratio (left column) and Maximum likelihood SEM (right column). The parameters used in each simulation are displayed in the legend in the top left (or bottom right) corner of each graph. The power of the Wald method to detect a positive causal effect of the exposure on the outcome increases slightly with increasing (residual) correlation between y_1_ and y_2_, whereas power remains static in the case of SEM

Both the Wald method and fitting the SEM yielded accurate estimates of the ${\beta }_{21}$ causal effect that were very similar (Supplementary Fig. 1, Supplementary Fig. 2) and were roughly comparable in terms of standard errors (Supplementary Fig. 1, Supplementary Fig. 3), statistical power (Supplementary Fig. 4), type I error rate (Supplementary Fig. 5) and coverage (Supplementary Fig. 6) (Supplementary Table 1). An exception was power to detect ${\beta }_{21}$ when the second instrument (x_2_) explained large portions of the variance in y_2_ (i.e. ${\gamma }_{22}$ was large). In this case, the SEM often yielded estimates of ${\beta }_{21}$ that had smaller standard errors and hence had greater power to detect this causal parameter (Fig. 2) (similar results were observed in the case of ${\beta }_{12}$ and increasing variance explained by ${\gamma }_{11}$—data not shown). Interestingly, the power of the Wald method/2SLS to detect a positive (negative) causal effect of the exposure on the outcome increased slightly with increasing (decreasing) residual correlation between y_1_ and y_2_, whereas power remained static in the case of SEM (Fig. 3).

Biology is complicated! Many physiological systems involve feedback mechanisms (e.g. to maintain homeostasis) (Zheng et al. 2023) and yet feedback loops are not typically explicitly modelled in Mendelian randomization studies. Nevertheless, our asymptotic derivations and finite sample simulations suggest that when the true underlying model involves a simple linear bidirectional feedback loop between two normally distributed quantitative variables, there is little difference in performance between traditional IV estimators and maximum likelihood structural equation modelling. This statement assumes that MR is performed in “both directions” if the Wald or two-stage least squares estimator is being used—a process referred to as reciprocal MR or bidirectional MR in the literature (Timpson et al. 2011). Both estimators produce consistent estimates of causal effects, although fitting the SEM does yield a small advantage in terms of power to detect ${\beta }_{21}$ when the second instrument explains a large proportion of variance in the outcome variable (or equivalently power to detect ${\beta }_{12}$ when x_1_ explains a significant proportion of the variance in y_1_). Intuitively, this is because the modelling of x_2_ “explains” some of the “error” variance in the outcome. We note that this advantage of SEM could be offset by first regressing y_2_ on x_2_ and then using the Wald estimator to estimate the causal effect of y_1_ on the residuals of this regression (and similarly in the other direction).

Interestingly, the Wald estimator/2SLS often showed slightly greater power to detect a positive (negative) causal effect of the exposure on the outcome compared to SEM as the residual correlation between y_1_ and y_2_ increased (decreased). It is well known that in the case of simple MR analyses with one genetic instrument, one exposure, one outcome and no feedback loops, the power of the Wald estimator/2SLS improves with increased residual covariance between the exposure and outcome (Brion et al. 2013). This can be appreciated by examining the formula for the approximate standard error of the Wald estimator (as derived by the Delta method (Thomas et al. 2007)):

$$\text{var}\left({\widehat{\beta }}^{*}\right)\cong \frac{\text{var}\left({\widehat{\beta }}_{ZY}\right)}{{\widehat{\beta }}_{ZX}^{2}}+\frac{{\widehat{\beta }}_{ZY}^{2}}{{\widehat{\beta }}_{ZX}^{4}}\text{var}\left({\widehat{\beta }}_{ZX}\right)-2\frac{{\widehat{\beta }}_{ZY}}{{\widehat{\beta }}_{ZX}^{3}}\text{cov}\left({\widehat{\beta }}_{ZX},{\widehat{\beta }}_{ZY}\right)$$

where ${\widehat{\beta }}^{*}$ is the Wald estimator of the causal effect of exposure on outcome, ${\widehat{\beta }}_{ZY}$ the regression coefficient for the SNP outcome association, and ${\widehat{\beta }}_{ZX}$ the regression coefficient for the SNP exposure association. The final term in this expansion involving the covariance between SNP-exposure and SNP-outcome regression coefficients, shows that the variance of the estimator should decrease as the residual correlation between exposure and outcome in overlapping individuals increases (assuming ${\widehat{\beta }}_{ZY}$ and ${\widehat{\beta }}_{ZX}$ are positive). In contrast, the residual covariance does not affect the power to detect a causal effect when using SEM. Intuitively, the reason is that an extra degree of freedom is expended modelling this covariance term in the SEM. In other words, SEM provides an estimate of this residual covariance, but at the expense of potentially decreased power to detect a causal effect when the residual covariance is in the same direction and similar or greater magnitude as the causal effect. The flipside is that SEM can potentially offer slightly better power than the Wald ratio/2SLS when the residual covariance is in the opposite direction to the estimated causal effect.

Our results assume strong valid instruments, continuous endogenous variables (y_1_, y_2_), linear relationships between all variables, no effect modification, normally distributed errors for y_1_ and y_2_ and that the causal influence of y_1_ on y_2_ (and vice versa) is due to a simple linear feedback loop. Our results also do not necessarily generalize to more complicated models involving more than two outcome variables (i.e. complex networks consisting of many different variables and feedback loops (Burgess et al. 2015)) where structural equation modelling may have advantages over simpler estimators. Indeed, in the Supplementary Note we provide an example of a more complicated SEM where the Wald Estimator does not yield consistent estimates of the true causal parameters. In these more complicated situations, whilst it may be possible to cobble together a consistent estimator using e.g. a combination of Wald ratios, to do so would be tedious, error prone and estimating the standard errors of such estimates may be difficult.

We also note that SEM provides an elegant way of modelling related individuals within the context of MR studies and facility to concurrently estimate other population parameters that might be of interest (e.g. underlying genetic and environmental variance components Castro-de-Araujo et al. 2023; Minica et al. 2018)). It is also straightforward within the SEM framework to model the effect of moderator and/or mediator variables, whereas this can be challenging for all but the simplest of situations using the traditional instrumental variables estimators (Relton and Davey Smith 2012). Finally, we note that more recent extensions to the MR paradigm explicitly consider the possibility of bidirectional relationships and in some cases attempt to model their effects (Brown and Knowles 2021; Hemani et al. 2017).

As the size of genome-wide association studies continues to increase, and potential genetic instruments become more ubiquitous, it will be interesting to see whether feedback loops of the sort described in this manuscript can be robustly detected. Our results suggest that both SEMs and traditional instrumental variables estimators will be suitable for identifying potential feedback loops, at least for the simple scenarios that we have investigated. For more complicated situations involving mediation, moderation and/or related individuals, the SEM framework provides considerable flexibility and a number of potential advantages over traditional instrumental variables estimators.

### Supplementary Information

Below is the link to the electronic supplementary material.

Supplementary file1 (PNG 713 KB)

Supplementary file2 (PNG 460 KB)

Supplementary file3 (PNG 393 KB)

Supplementary file4 (PNG 171 KB)

Supplementary file5 (PNG 166 KB)

Supplementary file6 (DOCX 84 KB)

Supplementary file7 (XLSX 248 KB)
