# Supplementary figures and images for: A Note on Modelling Bidirectional Feedback Loops in Mendelian Randomization Studies
Source: Behav Genet. 2024 May 31;54(4):367–73. doi: 10.1007/s10519-024-10183-0 (PMC11196367; doi:10.1007/s10519-024-10183-0)

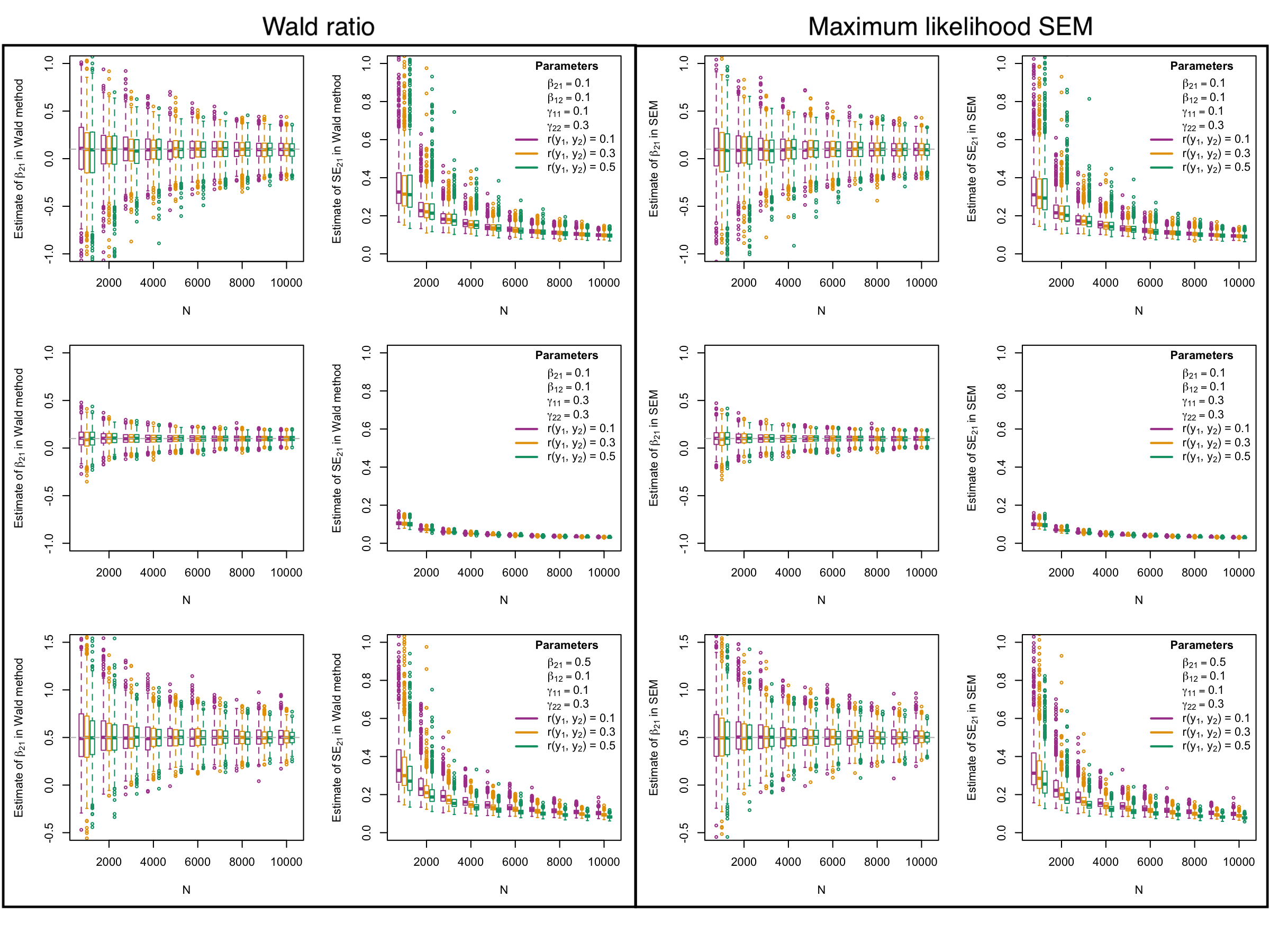

Supplement: Supplementary file 1 — Supplementary file1 (PNG 713 KB) [file 10519_2024_10183_MOESM1_ESM.png]

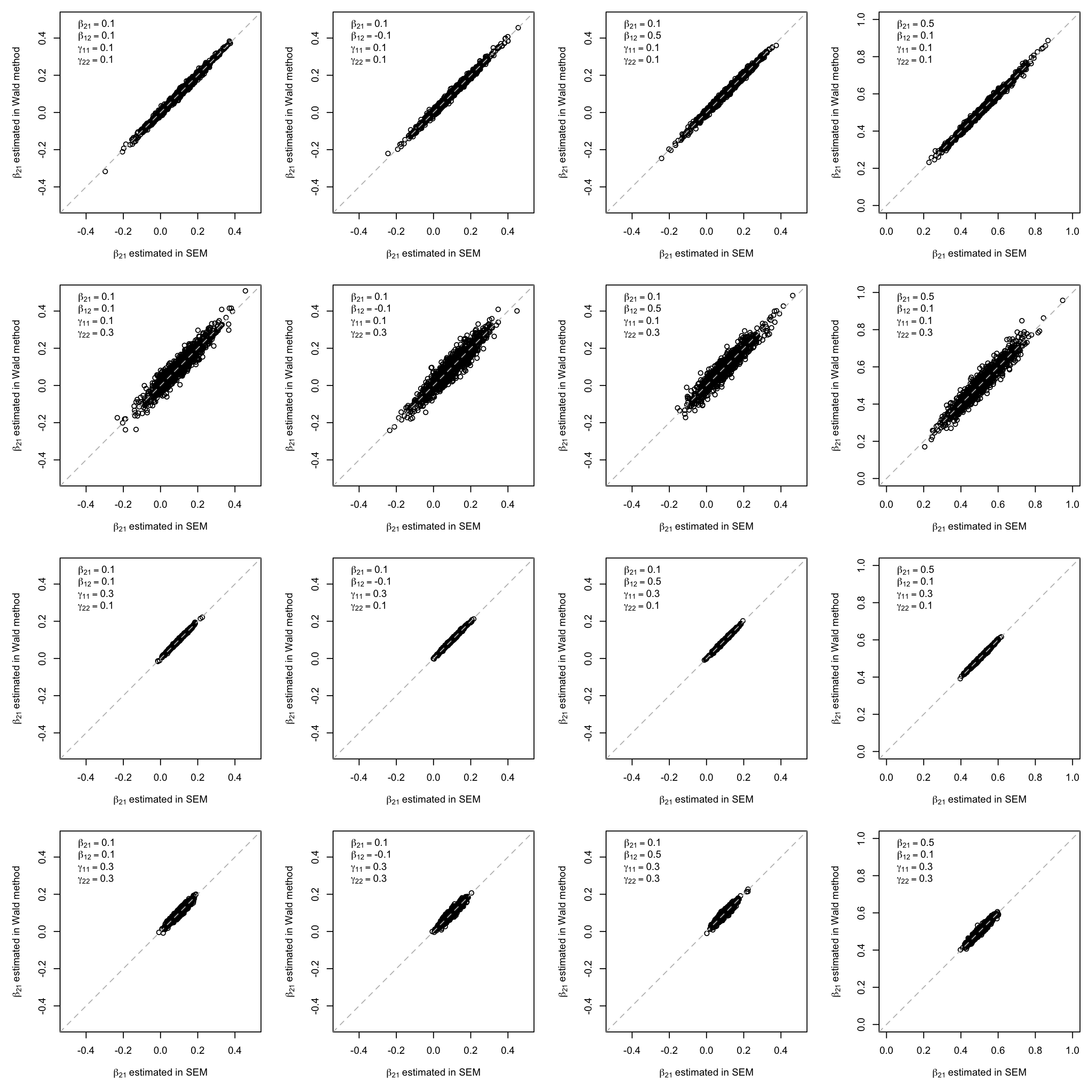

Supplement: Supplementary file 2 — Supplementary file2 (PNG 460 KB) [file 10519_2024_10183_MOESM2_ESM.png]

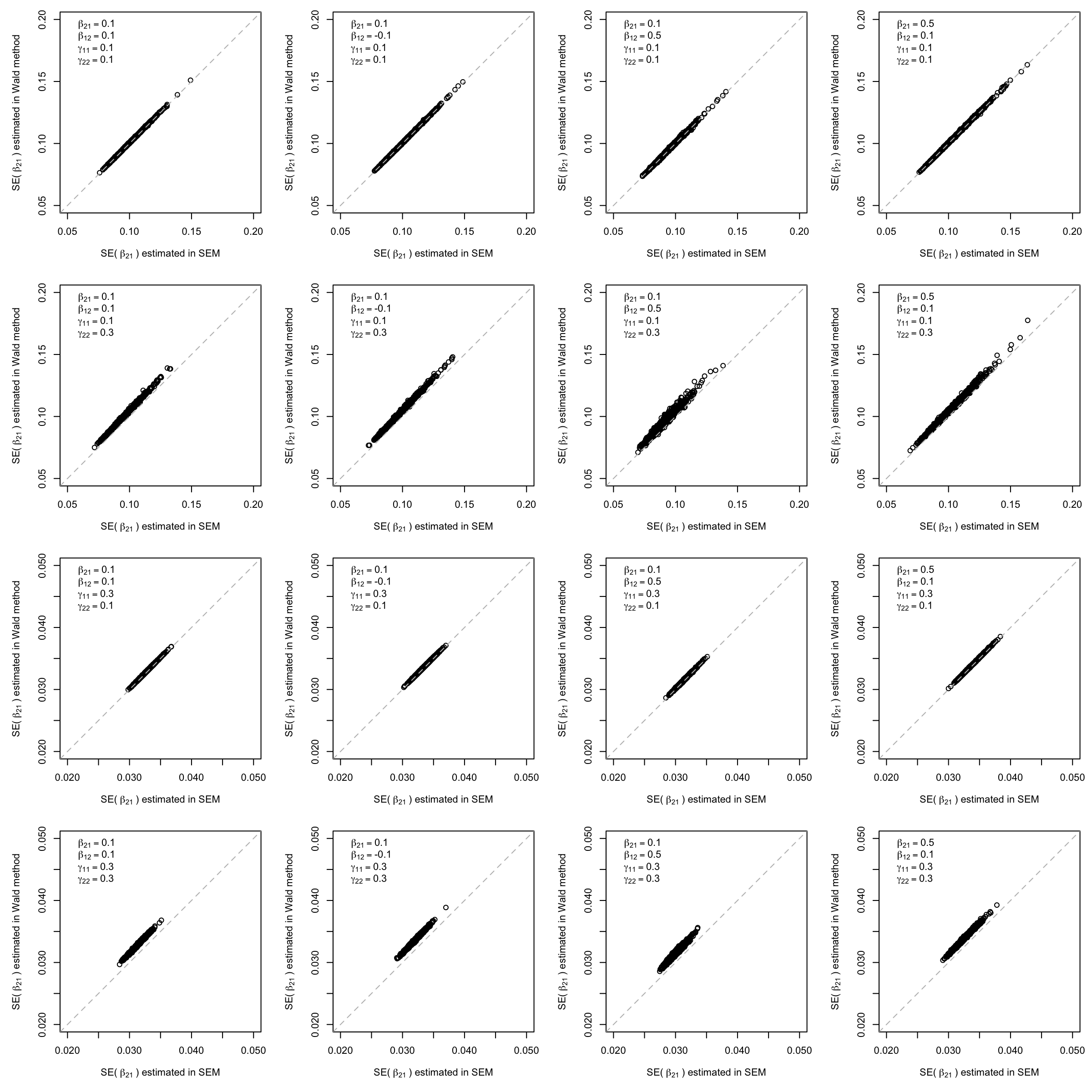

Supplement: Supplementary file 3 — Supplementary file3 (PNG 393 KB) [file 10519_2024_10183_MOESM3_ESM.png]

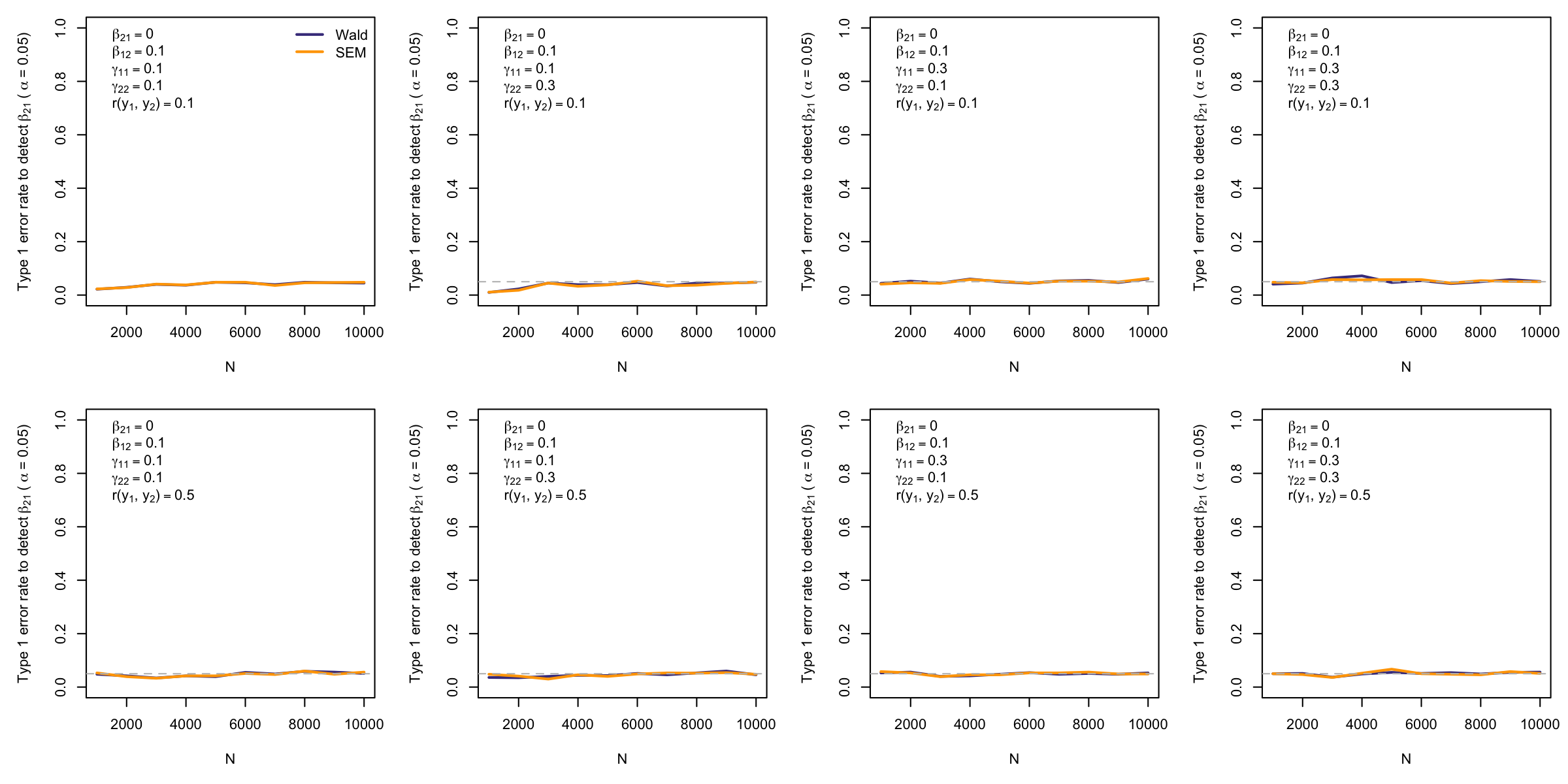

Supplement: Supplementary file 4 — Supplementary file4 (PNG 171 KB) [file 10519_2024_10183_MOESM4_ESM.png]

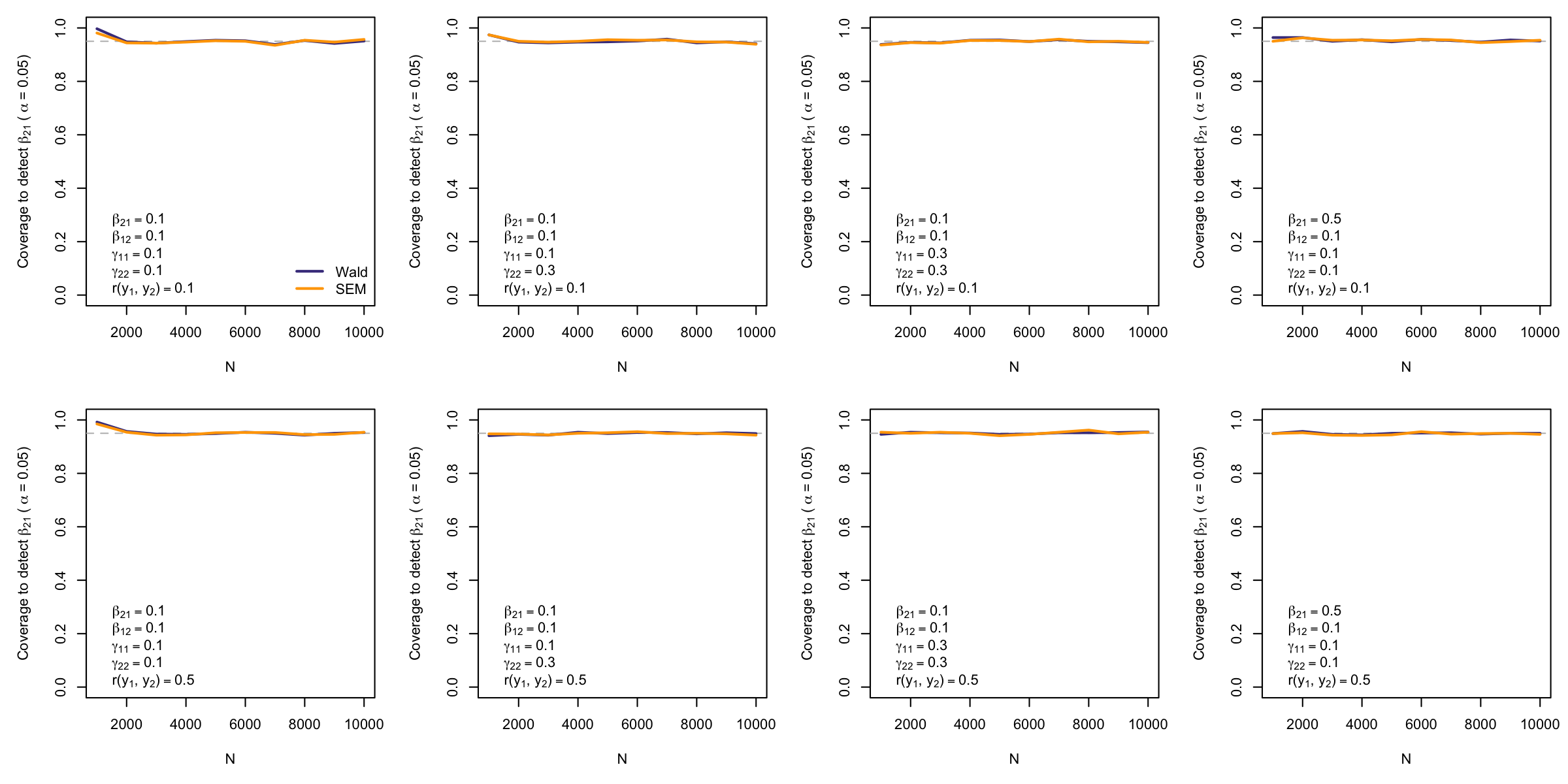

Supplement: Supplementary file 5 — Supplementary file5 (PNG 166 KB) [file 10519_2024_10183_MOESM5_ESM.png]
