# Supplementary material for: A Note on Modelling Bidirectional Feedback Loops in Mendelian Randomization Studies
Source: Behav Genet. 2024 May 31;54(4):367–73. doi: 10.1007/s10519-024-10183-0 (PMC11196367; doi:10.1007/s10519-024-10183-0)
Supplement: Supplementary file 6 — Supplementary file6 (DOCX 84 KB) [file 10519_2024_10183_MOESM6_ESM.docx]

**Correlated instruments bivariate case**

We examine the consistency of the Wald estimator in the bivariate case where the instruments may be correlated. This corresponds to the population model below.


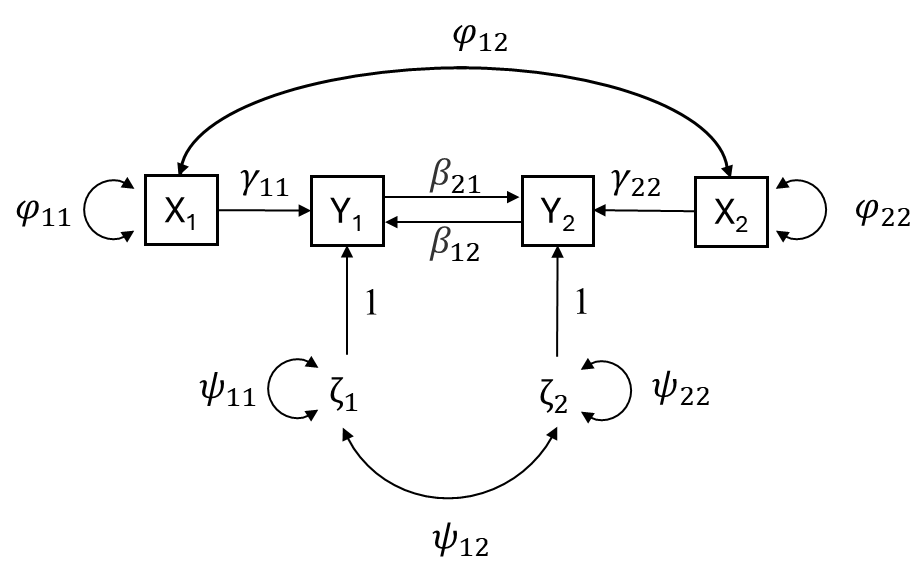


**Supplementary Figure 7.** Bidirectional feedback loop model with correlated instruments.

Taking the probability limit of the Wald estimator ($\hat{\beta}_{21}^{*}$) for the causal effect of y_1_ on y_2_ yields:

$$\mathrm{plim}\left( \hat{\beta}_{21}^{*} \right)=\mathrm{plim}\left[ \frac{\mathrm{cov}(x_{1}, y_{2})}{\mathrm{cov}(x_{1}, y_{1})} \right]=\frac{\mathrm{COV}(x_{1}, y_{2})}{\mathrm{COV}(x_{1}, y_{1})}=\frac{\phi_{11}\gamma_{11}\beta_{21}+\phi_{12}\gamma_{22}}{\phi_{11}\gamma_{11}+\phi_{12}\gamma_{22}\beta_{12}}\neq\beta_{21}$$

A similar calculation shows that the Wald estimator for the causal effect of Y_2_ on Y_1_ $(\hat{\beta}_{12}^{*}$) is also inconsistent.

**Reciprocal causal model for three dependent variables**

Consider the complicated population model given in Supplementary Figure 7 below. The three endogenous variables of interest (y_1_, y_2_, y_3_) are each proxied by three exogenous instruments (x_1_, x_2_, x_3_). Bidirectional feedback loops exist between all the y variables, as well as residual covariances (i.e. through confounding). Such a network might be an appropriate model for e.g. dietary intake of protein, carbohydrates and fat, where we would expect that an increase in consumption of one component would lead to a decrease in consumption of the other constituents. We first introduce the model in LISREL notation. Second, we show that the model is identified. Finally, we show that the Wald estimator does not produce consistent estimates of the causal effect parameters (i.e. β_12_, β_21_, β_13_, β_31_, β_32_, β_23_).


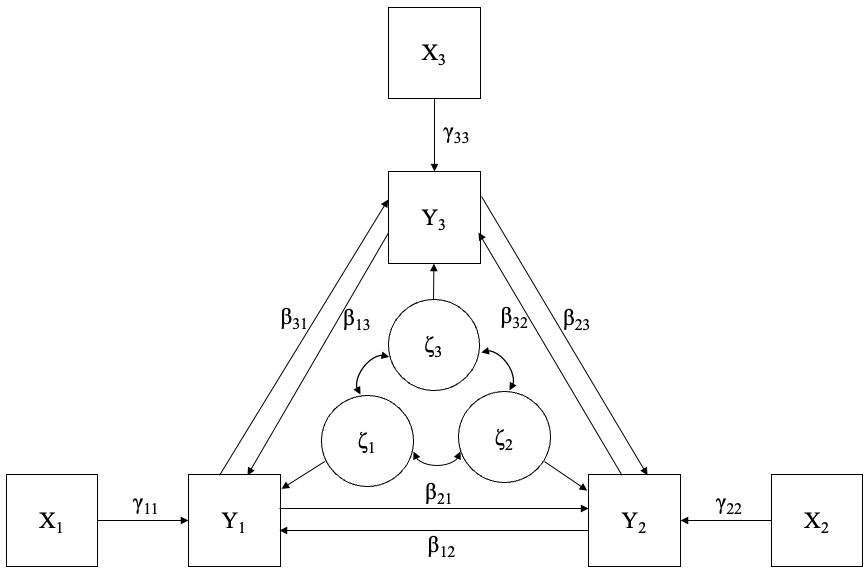


**Supplementary Figure 8.** A bidirectional feedback model involving three correlated endogenous variables and three independent instruments for them.

Model specification in LISREL matrix notation

$$\mathbf{y}= \mathbf{By}+\boldsymbol{\Gamma x}+\boldsymbol{\zeta}$$

where

$\mathbf{y}=\left[ \begin{matrix} y_{1} \\ y_{2} \\ y_{3} \end{matrix} \right]$; 3 x 1 vector of observed dependent variables

$\mathbf{x}=\left[ \begin{matrix} x_{1} \\ x_{2} \\ x_{3} \end{matrix} \right]$; 3 x 1 vector of SNP instruments

$\mathbf{B}=\left[ \begin{matrix} 0 & \beta_{12} & \beta_{13} \\ \beta_{21} & 0 & \beta_{23} \\ \beta_{31} & \beta_{32} & 0 \end{matrix} \right]$; 3 x 3 coefficient matrix for the causal effects of the **y** variables

$\boldsymbol{\Gamma}=\left[ \begin{matrix} \gamma_{11} & 0 & 0 \\ 0 & \gamma_{22} & 0 \\ 0 & 0 & \gamma_{33} \end{matrix} \right]$; 3 x 3 coefficient matrix for the SNP effects of the **x** on **y** variables

$\boldsymbol{\zeta}=\left[ \begin{matrix} \zeta_{1} \\ \zeta_{2} \\ \zeta_{3} \end{matrix} \right]$; 3 x 1 vector of errors

$\boldsymbol{\Phi}=\left[ \begin{matrix} \phi_{11} & 0 & 0 \\ 0 & \phi_{22} & 0 \\ 0 & 0 & \phi_{33} \end{matrix} \right]$ is the covariance matrix of **x**

$\boldsymbol{\Psi}=\left[ \begin{matrix} \psi_{11} & & \\ \psi_{21} & \psi_{22} & \\ \psi_{31} & \psi_{32} & \psi_{33} \end{matrix} \right]$ is the covariance matrix of residual errors **ζ** (lower elements only)

Identification of reciprocal causal model for three dependent variables

We use the “rank condition rule” (Bollen, 1989 p. 101) to establish the identification status of the model. The rank rule starts with creating the matrix **C**:

$$\mathbf{C}\boldsymbol{=}\left[ \boldsymbol{(}\mathbf{I-B}\boldsymbol{)} \right.\boldsymbol{|}\left. \boldsymbol{-\Gamma} \right]=\left[ \begin{matrix} 1 & {-\beta}_{12} & {-\beta}_{13} \\ {-\beta}_{21} & 1 & {-\beta}_{23} \\ {-\beta}_{31} & {-\beta}_{32} & 1 \end{matrix} \right.\left. \begin{matrix} {-\gamma}_{11} & 0 & 0 \\ 0 & {-\gamma}_{22} & 0 \\ 0 & 0 & {-\gamma}_{33} \end{matrix} \right]$$

To check the identification status of the *i*th equation, we delete all columns of **C** that do not have zeros in the *i*th row of **C**. We then use the remaining columns to form a new matrix, **C_i_**. A necessary and sufficient condition for the identification of the *i*th equation is that the rank of **C_i_** equals $(p-1\mathbf{)}$, where p is the number of y variables (so in this case two):

$\mathbf{C}_{\boldsymbol{1}}\mathbf{=}\left[ \begin{matrix} 0 & 0 \\ {-\gamma}_{22} & 0 \\ 0 & {-\gamma}_{33} \end{matrix} \right]$; rank = 2

$\mathbf{C}_{\boldsymbol{2}}\mathbf{=}\left[ \begin{matrix} {-\gamma}_{11} & 0 \\ 0 & 0 \\ 0 & {-\gamma}_{33} \end{matrix} \right]$; rank = 2

$\mathbf{C}_{\boldsymbol{3}}\mathbf{=}\left[ \begin{matrix} {-\gamma}_{11} & 0 \\ 0 & {-\gamma}_{22} \\ 0 & 0 \end{matrix} \right]$; rank = 2

Since all equations are identified, the model as a whole is identified.

(In)consistency of the Wald and 2SLS estimators

We calculate probability limits for the Wald ratio estimator for each causal parameter:

$$\mathrm{plim}\left( \hat{\beta}_{21}^{*} \right)=\mathrm{plim}\left[ \frac{\mathrm{cov}(x_{1}, y_{2})}{\mathrm{cov}(x_{1}, y_{1})} \right]=\frac{\mathrm{COV}(x_{1}, y_{2})}{\mathrm{COV}(x_{1}, y_{1})} =\frac{\beta_{23}\beta_{31}+\beta_{21}}{1-\beta_{23}\beta_{32}}\neq\beta_{21}$$

$$\mathrm{plim}\left( \hat{\beta}_{31}^{*} \right)=\mathrm{plim}\left[ \frac{\mathrm{cov}(x_{1}, y_{3})}{\mathrm{cov}(x_{1}, y_{1})} \right]=\frac{\mathrm{COV}(x_{1}, y_{3})}{\mathrm{COV}(x_{1}, y_{1})} =\frac{\beta_{21}\beta_{32}+\beta_{31}}{1-\beta_{23}\beta_{32}} \neq\beta_{31}$$

$$\mathrm{plim}\left( \hat{\beta}_{12}^{*} \right)=\mathrm{plim}\left[ \frac{\mathrm{cov}(x_{2}, y_{1})}{\mathrm{cov}(x_{2}, y_{2})} \right]=\frac{\mathrm{COV}(x_{2}, y_{1})}{\mathrm{COV}(x_{2}, y_{2})} =\frac{\beta_{13}\beta_{32}+\beta_{12}}{1-\beta_{13}\beta_{31}}\neq\beta_{12}$$

$$\mathrm{plim}\left( \hat{\beta}_{32}^{*} \right)=\mathrm{plim}\left[ \frac{\mathrm{cov}(x_{2}, y_{3})}{\mathrm{cov}(x_{2}, y_{2})} \right]=\frac{\mathrm{COV}(x_{2}, y_{3})}{\mathrm{COV}(x_{2}, y_{2})} =\frac{\beta_{12}\beta_{31}+\beta_{32}}{1-\beta_{13}\beta_{31}}\neq\beta_{32}$$

$$\mathrm{plim}\left( \hat{\beta}_{13}^{*} \right)=\mathrm{plim}\left[ \frac{\mathrm{cov}(x_{3}, y_{1})}{\mathrm{cov}(x_{3}, y_{3})} \right]=\frac{\mathrm{COV}(x_{3}, y_{1})}{\mathrm{COV}(x_{3}, y_{3})} =\frac{\beta_{12}\beta_{23}+\beta_{13}}{{1-\beta}_{12}\beta_{21}}\neq\beta_{13}$$

$$\mathrm{plim}\left( \hat{\beta}_{23}^{*} \right)=\mathrm{plim}\left[ \frac{\mathrm{cov}(x_{3}, y_{2})}{\mathrm{cov}(x_{3}, y_{3})} \right]=\frac{\mathrm{COV}(x_{3}, y_{2})}{\mathrm{COV}(x_{3}, y_{3})} =\frac{\beta_{13}\beta_{21}+\beta_{23}}{{1-\beta}_{12}\beta_{21}}\neq\beta_{23}$$

These calculations show that the Wald ratio estimates are inconsistent estimates of the relevant causal parameters.

**References**

Bollen, K. (1989). *Structural equations with latent variables*. Hoboken, NJ, USA: John Wiley and Sons Inc
